# The Domestication Syndrome in *Phoenix dactylifera* Seeds: Toward the Identification of Wild Date Palm Populations

**DOI:** 10.1371/journal.pone.0152394

**Published:** 2016-03-24

**Authors:** Muriel Gros-Balthazard, Claire Newton, Sarah Ivorra, Marie-Hélène Pierre, Jean-Christophe Pintaud, Jean-Frédéric Terral

**Affiliations:** 1 Institut des Sciences de l’Evolution, Université - Montpellier, UMR 5554 CNRS / Université de Montpellier / IRD / EPHE, CC065, Equipe Dynamique de la Biodiversité, Anthropo-écologie, Place Eugène Bataillon, 34095, Montpellier Cedex 5, France; 2 UMR DIADE, équipe DYNADIV, Institut de Recherche pour le Développement, 911 avenue Agropolis, 34394, Montpellier cedex 5, France; 3 Laboratoire d’Archéologie et de Patrimoine, Université du Québec à Rimouski, 300 Allée des Ursulines, Rimouski (Qc), G5L 3AI, Canada; Washington University, UNITED STATES

## Abstract

Investigating crop origins is a priority to understand the evolution of plants under domestication, develop strategies for conservation and valorization of agrobiodiversity and acquire fundamental knowledge for cultivar improvement. The date palm (*Phoenix dactylifera* L.) belongs to the genus *Phoenix*, which comprises 14 species morphologically very close, sometimes hardly distinguishable. It has been cultivated for millennia in the Middle East and in North Africa and constitutes the keystone of oasis agriculture. Yet, its origins remain poorly understood as no wild populations are identified. Uncultivated populations have been described but they might represent feral, i.e. formerly cultivated, abandoned forms rather than truly wild populations. In this context, this study based on morphometrics applied to 1625 *Phoenix* seeds aims to (1) differentiate *Phoenix* species and (2) depict the domestication syndrome observed in cultivated date palm seeds using other *Phoenix* species as a “wild” reference. This will help discriminate truly wild from feral forms, thus providing new insights into the evolutionary history of this species. Seed size was evaluated using four parameters: length, width, thickness and dorsal view surface. Seed shape was quantified using outline analyses based on the Elliptic Fourier Transform method. The size and shape of seeds allowed an accurate differentiation of *Phoenix* species. The cultivated date palm shows distinctive size and shape features, compared to other *Phoenix* species: seeds are longer and elongated. This morphological shift may be interpreted as a domestication syndrome, resulting from the long-term history of cultivation, selection and human-mediated dispersion. Based on seed attributes, some uncultivated date palms from Oman may be identified as wild. This opens new prospects regarding the possible existence and characterization of relict wild populations and consequently for the understanding of the date palm origins. Finally, we here describe a pipeline for the identification of the domestication syndrome in seeds that could be used in other crops.

## Introduction

Global food security is facing challenges posed by the sharp reduction in the diversity of cultivated plants associated with planetary changes and the increasing food demand [1–4]. The implementation of crop improvement programs is expected to boost food production and food security and thus to help rising to the current and future challenges of crop cultivation. The identification of wild populations is one of the important prerequisites for breeding programs, as it is long known that they represent a genetic resource for cultivar improvement [5]. Moreover, it opens the possibility of comparing wild and domesticates to identify selected traits and understand evolution patterns of phenotypic traits, some of them defining the domestication syndrome [6–12]. For most crops, especially annuals, the wild ancestor is well known and populations are characterized, so that their domestication histories have been intensively studied [7–9]. In perennials, in contrast, we have a much less comprehensive knowledge due to their long life, ongoing crop-wild gene flow and clonal propagation that contribute to mild domestication bottlenecks and thus weak domestication syndrome [13,14]. Escaped individuals (called feral) from more or less distant cultivation areas may survive and reproduce without human intervention (e.g. olive tree [15] and grapevine [16]). It is therefore difficult to identify truly wild populations, as demonstrated in olive trees [17]. More strikingly, in date palms (*Phoenix dactylifera* L., Arecaceae) no wild population has been characterized to date [18–20].

The date palm belongs to the Old World genus *Phoenix* L. (Arecaceae) composed of 14 inter-fertile species distributed from the Atlantic islands, through Southern Europe, Africa and Southern Asia to the Philippines [21,22]. The whole genus is economically very important as most species are cultivated or exploited for many purposes such as ornamentation, food or construction. Recent barcoding studies based on nuclear and chloroplastic sequences allowed to identify unambiguously nine of the 13 *Phoenix* species included and identified the date palm sister species as *Phoenix sylvestris* and *Phoenix atlantica* [18,23,24]. However, *Phoenix* species are morphologically close and sometimes hardly distinguishable as there are only few systematically useful morphological and anatomical characters [18,21]. Additional features are therefore required to easily distinguish *Phoenix* species.

The most important species of the genus, the date palm, constitutes the main element in oasis agro-ecosystems and has assumed a nutritional, economic and symbolic role for millennia [25]. It not only provides dates, a highly nutritious fruit [26], but it also allows the cultivation of other crops by protecting them from sun, heat and wind: this is the oasis polyculture system [27].

Traditional areas of cultivation are North Africa and the Middle East stretching as far as Pakistan and North-Western India [21]. In recent centuries, it was introduced in America, sub-Saharan Africa, Southern Europe and Oceania as a fruit crop or for ornamental and religious purposes [21]. Despite the importance of its cultivation, we possess little data about the date palm origins of domestication, historical biogeography and evolutionary history. According to archaeological data, date palm cultivation, also known as phoeniciculture, seems to emerge between the 5^th^ and the 3^rd^ millennium BC in the Middle East, more precisely around the Persian Gulf [25]. The cultivated date palm derives from wild populations of the same species, but in the current state of research none is securely identified [18,19]. Indeed, spontaneously growing or uncultivated populations are found within its whole distribution area [28,29] but no tangible element to differentiate wild from feral date palms has been evidenced [28]. Therefore, the status of the mentioned uncultivated date palm populations remains to be clarified.

Traditional (study of size) and geometric morphometrics (outline analysis) applied to seeds appear as two attractive and complementary tools to differentiate distinct species [30–34], distinguish wild from domesticated crops (e.g. in the olive tree [17,35], grapevine [36] or caimito [37]) and detect or suspect feral individuals [17,38]. Focusing on seeds rather than other plant organs is interesting because seeds are easily sampled and stored, and keep well. Very interestingly, they are intimately related to the fruit, i.e. the main object of selection in a fruit crop like the date palm, since the increase in seed size is likely linked allometrically to increase in fruit size [39–41]. In addition, seeds are the most abundant archaeological remains reflecting, in Egypt and the Persian Gulf area, the traditional use of date palm for over 6,000 years [25] and can thus be used to study past agrobiodiversity and the emergence of cultivation [38,42]. *Phoenix* seeds display a hard endosperm and are characterized by a deeply grooved raphe [21]. Seeds of *Phoenix* have been previously described [19,21,43,44]. They are of varying size and shape [19,21,43,44]. Length ranges from 7 mm in *Phoenix roebelenii* to 30 mm in cultivated date palm [21]. They are elongated in date palm cultivars while they are rounded in other *Phoenix* species [19]. Nevertheless, a comprehensive study combining size and shape analysis of *Phoenix* seeds is still required. Indeed, the aforementioned studies use qualitative descriptors for size and shape or focus only on size or shape rather than combining both information types. In addition, a recent study suggests the possible wild status of some date palm individuals spontaneously growing in Oman, based on the wild morphotype of their seeds [19]. Thus, the capability of distinguishing feral, wild and cultivated date palms based on seed morphology needs to be carefully assessed as it represents a major challenge in the understanding of date palm domestication history.

The objective of this study is to improve our knowledge of the origins of the cultivated date palm and of the morphological changes that occurred under domestication, i.e. infer the domestication syndrome affecting the seeds. A morphometric study of seeds belonging to different *Phoenix* species was carried out. Firstly, it aimed at evaluating the potentiality of seed size and shape to distinguish *Phoenix* species. Secondly, seed comparison between cultivated date palms and other *Phoenix* species was expected to help predicting seed size and shape in wild date palms. Because morphology expresses an essential part of the phenotype, it is an important indicator of the nature of selection pressures, including environmental constraints and anthropogenic factors. In the case of wild *Phoenix* species, as in that of wild date palm, the environmental context including both abiotic and biotic factors represents a set of natural selection pressures, while size and shape of cultivated date palm seeds, subjected to artificial selection, were differentiated under domestication. As a consequence, we used *Phoenix* non *dactylifera* species as a wild reference to anticipate seed size and shape in wild date palms. Our results first demonstrate that it is possible to differentiate most *Phoenix* species based on their seed size and shape and that seed morphometrics is a reliable tool to corroborate the species delimitation of the *Phoenix* species previously derived from nuclear and chloroplastic data [18,23,24]. We showed that the cultivated date palms have distinct seed features compared to wild *Phoenix* species and that we expect feral and wild date palms to have different phenotypes. Based on this, the uncultivated samples from Oman included in this analysis could be truly wild date palms.

## Materials and Methods

### Sampling

#### Collection of *Phoenix* seeds

Seeds of 13 *Phoenix* species (all species of the genus except *P*. *atlantica*) were analysed in this work (Table 1; Table 2).

**Table 1:** *Phoenix dactylifera* seed samples for morphometric analyses. When different from the country of sampling, the country of origin of the cultivar is given in parenthesis. Acc. Nb.: Accession number; Nb. seed: Number of seeds.

Cultivar name	Acc. Nb.	Country of sampling (Origin)	Sampling authorized by	Nb. seed
**La Confitera**	0081_CON1	Spain	Estación Phoenix	20
**Iberica**	0072_IBE4	Spain	Estación Phoenix	20
**Bou Feggous**	0076_BFE1	Spain (Morocco)	Estación Phoenix	20
**Medjoul**	0083_MED1	Spain (Morocco)	Estación Phoenix	20
**Thorry**	0080_THO1	Spain (Algeria)	Estación Phoenix	20
**Ghars Mettig**	0212_GME2	Tunisia (Algeria)	Centre de Recherches sur l’Elevage et le Pâturage, Kébili	20
**Ahmar**	1249_AHM4	Mauritania	Market	20
**Tijib**	1254_TIJ2	Mauritania	Market	20
**Deglet Nour**	0186_DEG2	Tunisia	Centre Régional de Recherches sur l’Agriculture d’Oasis (Ministry of Agriculture), Degache	20
**Lagou**	0216_LAG2	Tunisia	Private land	20
**Tiswin**	1550_TIS1	Libya	Market	20
**Digla**	1552_DIG1	Libya	Market	20
**Shelabi**	0097_SHE1	Syria (Egypt)	Market	20
**Siwi**	0007_SIW3	Egypt	Private land	20
**Ibrahimi**	0093_IBR1	Syria	Market	20
**Om Asal**	0094_OMA1	Syria	Market	20
**Halaoui**	0198_HAL2	Tunisia (Iraq)	Centre Régional de Recherches sur l’Agriculture d’Oasis (Ministry of Agriculture), Degache	20
**Zaydi**	0079_ZAY1	Spain (Iraq)	Estación Phoenix	20
**Khalass**	0077_KHA1	Spain (Saudi Arabia)	Estación Phoenix	11
**Qadi**	0095_QAD1	Syria (Saudi Arabia)	Market	20
**Nashu Al Khasba**	0122_NBA1	Oman	Wadi Qurayat Collection, Research Department of the Ministry of Agriculture	20
**Khasab**	0139_KAB1	Oman	Wadi Qurayat Collection, Research Department of the Ministry of Agriculture	20
**Mozafati**	1549_MAZ1	France (Iran)	Market	20
**Iswid**	0107_ISW1	Syria (Iran)	Market	20
**Seedling**	1601_DAC492	India	Private land	20
**Seedling**	1625_DAC514	India	Collected in the wild. No permission required.	20
**Feral**	2431-DAC832	Egypt	Collected in the wild. No permission required.	20
**Feral**	2433-DAC834	Egypt	Collected in the wild. No permission required.	20
**Uncultivated**	344-WILD63	Oman	Research Department of the Ministry of Agriculture	20
**Uncultivated**	403-WILD82	Oman	Research Department of the Ministry of Agriculture	20

**Table 2:** *Phoenix* non *dactylifera* seed samples for morphometric analyses. Acc. Nb.: Accession number; Nb. seed: Number of seeds.

Species	Acc. Nb.	Location of sampling (Origin)	Nb. seed.
***Phoenix acaulis***	1267_ACA4	Ordered via internet (India)	15
***Phoenix acaulis***	1720_ACA6	Millenium Seed Bank, Kew, UK (India)	20
***Phoenix acaulis***	1867_ACA7	Herbarium Palmarum, Florence, Italy (India)	20
***Phoenix acaulis***	1871-ACA8	Herbarium Palmarum, Florence, Italy (India)	7
***Phoenix andamanensis***	2139_AND2	Royal Botanic Garden, Kew, UK (North Andaman, India)	13
***Phoenix caespitosa***	1322_CAE3	Royal Botanic Garden, Kew, UK (Somalia)	20
***Phoenix caespitosa***	1878_CAE4	Centro Studi Erbario Tropicale, Florence, Italy (Somalia)	20
***Phoenix caespitosa***	1879_CAE5	Centro Studi Erbario Tropicale, Florence, Italy (Somalia)	20
***Phoenix canariensis***	0721_CAN8	Bordighera, Italy	20
***Phoenix canariensis***	0880_CAN37	San Remo, Italy	20
***Phoenix canariensis***	0092_CAN1	Palavas, France	20
***Phoenix canariensis***	1870_CAN62	Herbarium Palmarum, Florence, Italy (Canary Islands)	20
***Phoenix canariensis***	1875_CAN63	Seed reference collection, Florence, Italy (Canary Islands)	20
***Phoenix loureiroi* var. pedunculata**	1722_LOR12	Millenium Seed Bank, Kew, UK (India)	20
***Phoenix loureiroi* var. pedunculata**	1863_LOR14	Herbarium Palmarum, Florence, Italy (India)	20
***Phoenix loureiroi* var. pedunculata**	1864_LOR15	Herbarium Palmarum, Florence, Italy (India)	20
***Phoenix loureiroi* var. pedunculata**	2140_LOR17	Millenium Seed Bank, Kew, UK (Bhutan)	20
***Phoenix loureiroi* var. loureiroi**	1865_LOR16	Herbarium Palmarum, Florence, Italy (Batanes Islands, Philippines)	20
***Phoenix loureiroi* var. loureiroi**	2143_LOR18	Millenium Seed Bank, Kew, UK (Thailand)	20
***Phoenix paludosa***	1868_PAL4	Herbarium Palmarum, Florence, Italy	20
***Phoenix paludosa***	1869_PAL5	Herbarium Palmarum, Florence, Italy	20
***Phoenix paludosa***	1872_PAL6	Herbarium Palmarum, Florence, Italy (Vietnam)	10
***Phoenix paludosa***	2144_PAL7	Millenium Seed Bank, Kew, UK (Thailand)	20
***Phoenix pusilla***	1873_PUS5	Herbarium Palmarum, Florence, Italy	20
***Phoenix pusilla***	1874_PUS6	Herbarium Palmarum, Florence, Italy (Sri Lanka)	20
***Phoenix pusilla***	2141_PUS7	Millenium Seed Bank, Kew, UK (Sri Lanka)	20
***Phoenix pusilla***	2142_PUS8	Millenium Seed Bank, Kew, UK (India)	20
***Phoenix pusilla***	2145_PUS9	Millenium Seed Bank, Kew, UK (Sri Lanka)	20
***Phoenix reclinata***	0441_REC1	Madagascar	20
***Phoenix reclinata***	0443_REC2	Montpellier herbarium (Benin)	20
***Phoenix reclinata***	0766_REC14	San Remo, Italy	20
***Phoenix reclinata***	0771_REC15	San Remo, Italy	20
***Phoenix reclinata***	1321_REC40	Millenium Seed Bank, Kew, UK (Tanzania)	20
***Phoenix reclinata***	1719_REC42	Millenium Seed Bank, Kew, UK (Gabon)	20
***Phoenix roebelenii***	0906_ROE4	San Remo, Italy	20
***Phoenix rupicola***	1721_RUP10	Millenium Seed Bank, Kew, UK (India)	9
***Phoenix rupicola***	1866_RUP11	Herbarium Palmarum, Florence, Italy (India)	20
***Phoenix rupicola***	1876_RUP12	Seed reference collection, Florence, Italy (India)	20
***Phoenix rupicola***	1877_RUP13	Seed reference collection, Florence, Italy (India)	20
***Phoenix sylvestris***	1653_SYL20	Kathiawar peninsula, Gujarat, India	20
***Phoenix sylvestris***	1680_SYL47	Aravalli range, Rajasthan, India	20
***Phoenix sylvestris***	1688_SYL55	Aravalli range, Rajasthan, India	20
***Phoenix sylvestris***	1689_SYL56	Aravalli range, Rajasthan, India	20
***Phoenix sylvestris***	1696_SYL63	Marwar region, Rajasthan, India	20
***Phoenix sylvestris***	1702_SYL69	Marwar region, Rajasthan, India	20
***Phoenix sylvestris***	1709_SYL76	Udaipur district, Rajasthan, India	20
***Phoenix sylvestris***	1712_SYL79	Udaipur district, Rajasthan, India	20
***Phoenix sylvestris***	1714_SYL81	Udaipur district, Rajasthan, India	20
***Phoenix sylvestris***	1718_SYL85	Udaipur district, Rajasthan, India	20
***Phoenix theophrasti***	1461_THE19	Vai, Crete, Greece	20
***Phoenix theophrasti***	1462_THE20	Vai, Crete, Greece	20
***Phoenix theophrasti***	1478_THE36	Martsalos, Crete, Greece	20
***Phoenix theophrasti***	1479_THE37	Martsalos, Crete, Greece	20
***Phoenix theophrasti***	2146_THE82	Millenium Seed Bank, Kew, UK (Crete, Greece)	20

Samples belonging to *P*. *dactylifera* consist in 26 cultivated date palms among which 24 are cultivars (clones) and two are seedlings. They originate from 30 different countries spanning the whole date palm distribution. The origin of these samples is stated as the country of sampling for seedlings and the country where it supposedly originates for cultivars (i.e. although the Deglet Noor cultivar is grown in Arabia, it is well known that it originates from Tunisia). It is important to note that the seed shape of a cultivar is only slightly affected by environmental conditions as it was previously evidenced [19]. Indeed, the same cultivar grown in two different countries display more closely related seeds than other cultivars studied [19]. The utilization of samples grown in a different region than the country of natural origin is therefore not of concern for this study. Seeds from four uncultivated date palms were also included: two feral date palms from an ancient and abandoned Egyptian palm grove as well as two potentially wild date palms growing in Oman [19] (Table 1). Most samples were collected on private lands or in collections and for any location, the landowner or the authority responsible for the collection gave us permission. For samples bought on markets or collected in the wild, no permission was required as the date palm is not an endangered or protected species and the collection was not carried out in national parks or other protected areas.

Material from other *Phoenix* species mostly comes from approved herbaria and collections (Royal Botanic Gardens, Millennium Seed Bank, and Carpological Collection, Kew, UK; Herbarium Palmarum, seed reference collection, Centro Studi Erbario Tropicale, Firenze, Italy; Montpellier herbarium of the Institute of Botany, France) and each location issued a specific permission to sample for this study. The material from collections was photographed on site and the photographs were subsequently used in the analyses. Some seeds were however directly collected in the field and identified without any doubt (Table 2). In those cases, specific permission was not required as the collected species are not protected or endangered and the collection was not carried out in national parks or other protected areas. For samples collected in herbaria, the region/country of origin attributed was defined as the original country of origin as stated on the herbarium voucher and when the information was missing or ambiguous, the origin was set as missing. *Phoenix atlantica* is the only missing species in this analysis, despite our tremendous effort in sampling. Previously considered as feral date palms or the product of hybridization between several *Phoenix* species [21,45], it has been only recently recognized as a distinct species [45]. Given its close morphology to the date palm [45,46] and its only recent status as a distinct species, sampling this species would necessitate a careful examination of specimens on its endemic Islands, Cape Verde, rather than sampling in herbarium where *Phoenix atlantica* samples might be date palms or hybrids.

As previously reported, non *dactylifera Phoenix* species (Table 2) are used as a “wild” reference compared to cultivated date palms in order to infer morphometrical features of wild date palm seeds. Although these species may be used by Human, they are not subject to artificial selection (undomesticated) compared to cultivated date palms and thus represents a reliable “wild” reference. They are referred as the “wild” *Phoenix*. Seeds were photographed in both dorsal and lateral sides in order to appreciate the real three-dimensional shapes.Number of seeds analysed per individual for a reliable characterization of morphological features

The number of *Phoenix* seeds to be analysed for an optimal evaluation of intra-individual shape variation when using Fourier coefficients method has already been tested and set at 20 [19]. The sample size necessary to correctly assess the seed dimension for one individual has not been tested and was therefore assessed in this study with five individuals of different species. For each of these five accessions, we randomly sampled one to 30 seeds. For each of these 30 subsets of different sample size, the average of the four size parameters was calculated. The number of seeds required for studying the size parameters was evaluated as the minimum number of seeds required to stabilize the mean of the dimension parameters that is the minimum number of seeds from which the averages are stable.

### Describing seed size and shape using traditional and geometric morphometrics

#### Size analysis of seeds

**Fig 1:**
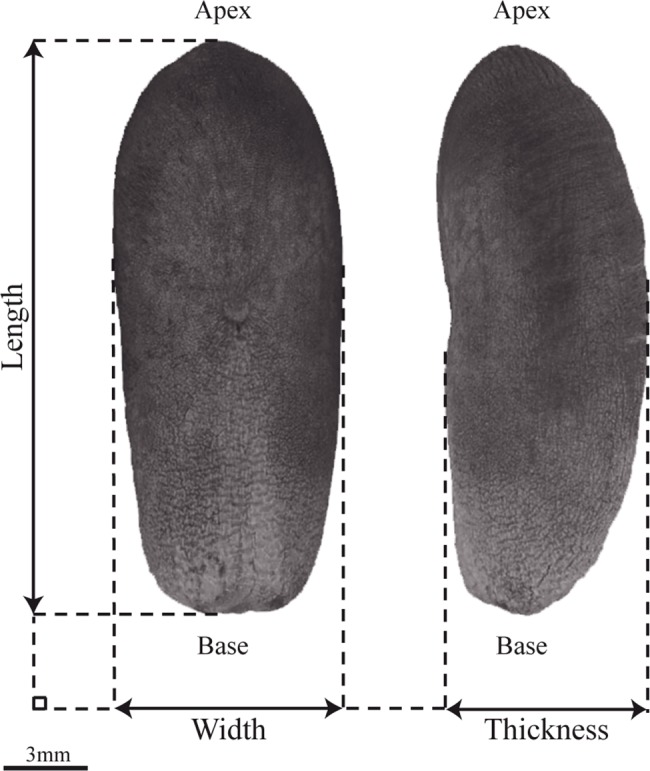
Overview of the seed size parameters measured in this study. Left: dorsal view; right: lateral view.

Four parameters representing the seed dimensions were measured using ImageJ version 1.42 [47] (Fig 1). Length and width of seeds were measured on the dorsal view. Thickness was measured as the maximum width of the seed lateral side. The surface of the dorsal side of the seed was also measured. The correlations between each pair of size parameters were plotted and assessed using Pearson product-moment correlation tests.

#### Fourier analysis of seed outlines

Seed shape was quantified using outline analyses based on Fourier method following the protocol developed on *Phoenix* seeds [19,48] and implemented in R software (Momocs package [49,50]). The x and y coordinates of 64 points equally spaced along the outline of each seed were extracted semi-automatically using an image analysis system, the starting-point being the seed base. Coordinates present a high quantity of redundant information and they therefore need to be standardised for size and orientation in order to retain shape information only [51]. For this purpose, they are transformed using the Elliptic Fourier Transform (EFT) method. It is based on the separate Fourier decomposition of the incremental changes of the x and y coordinates as a function of the cumulative length along the outline [48,52]. To each harmonic *n* correspond four coefficients: A_n_ and B_n_ for x, C_n_ and D_n_ for y, defining an xy-plane. In order to retain shape information mainly, the size is standardized and seeds are oriented using the coefficients of the best-fitting ellipse of any outline, that is the first harmonic (H1) [48].

The outline is described by a maximum of 32 harmonics (in case of an outline defined by 64 points) but the information added by each harmonic decreases with the rank of the harmonic while the measurement noise increases [53–55]. The number of harmonics for an optimal description of *Phoenix* seed outlines was evaluated as eight (H2 to H9 after the exclusion of H1 whose coefficients correspond to residuals after standardization) [19]. As a consequence, a set of 64 Fourier coefficients (i.e. four Fourier coefficients for each of the eight harmonics for both lateral and dorsal sides) was retained and exploited in the following statistical analysis.

### Discrimination of *Phoenix* species based on seed size and shape

The following statistical analyses were performed using the R software v2.15.3 [50].

#### Intra-specific variability of seed size and shape

For each species and each parameter of size, the range of values was computed as it represents the most obvious measure of variability. In order to visually inspect the variation of shape within species, a reconstruction of the mean seed outline of each sample was obtained using the inverse Fourier Transform method, following processes inverse to those used in calculating the Fourier coefficients [56].

Furthermore, the variability of size and shape within species was quantified by the dispersion of seed points around species centroid in two PCA (Principal Component Analysis, *dudi*.*pca* function) spaces; the first PCA was performed according to the four size parameters and the second according to the 64 Fourier coefficients related to both dorsal and lateral sides. For each species, the mean of the distances of seed points from the species centroid was computed. The distances were calculated as the sum of the squared distances in each PCA component, weighted by the variance explained by that component. Measure of intra-specific variability may be correlated with the number of seeds and individuals included, especially for small sample size. To standardize this measurement, we used the rarefaction method: a fixed number of seeds were randomly sampled one hundred times and the mean distance calculated over the one hundred replicates. This method allows quantification of the intra-specific variability among equal-sized samples drawn from the different species. The number of seeds to sample was evaluated at 20 (S2 Appendix). The intra-specific variability was thus calculated as the average of 100 mean distances between 20 randomly sampled seeds and the species centroid. Species represented by a single sample (*Phoenix andamanensis* and *P*. *roebelenii*) were excluded, as the calculation of intra-specific variability has no meaning in this case. The difference of variability of size and shape among species was tested with post-hoc Tukey’s test (*HSD*.*test* function).

#### Size and shape specificity of each *Phoenix* species

The homoscedasticity and the normality of each seed measurements were tested using Bartlett’s test and Shapiro-Wilk’s test respectively (*bartlett*.*test* and *shapiro*.*test* functions). The difference in seed dimensions between *Phoenix* species was tested using first nested ANOVAs (Analysis of variance) on each dimension parameter (*aov* function) with individual accession nested in species in order to take into account the non-independence of seeds and secondly post-hoc Tukey’s test. To evaluate the among-species differentiation of seed shape variation, a PCA was carried out on the 64 Fourier coefficients and the homoscedasticity and the normality of the coordinates were tested using Bartlett’s test and Shapiro-Wilk’s test respectively. A nested MANOVA (Multivariate analysis of variance, *manova* function) was performed on the first five coordinates, the explanatory variable being the species. To test the discrimination between the different *Phoenix* species in relation with the seed size and shape, three Linear Discriminant Analyses (LDA, *lda* function) were performed according to (1) size parameters, (2) 64 Fourier coefficients associated with both dorsal and lateral sides, (3) the combination of dimension parameters and 64 Fourier coefficients associated with both dorsal and lateral sides. To estimate the discriminant power of the LDAs, leave-one-out cross-validations were performed: posterior species assignations were executed for each seed (*lda* function with option CV = T). The discriminating rate of each species was calculated as the percentage of positive allocation.

### Seed comparison between cultivated date palms and “wild” *Phoenix*

The seed dimension was compared among 4 groups: cultivated date palms, feral date palms, Oman uncultivated date palms of unknown status and *Phoenix* non *dactylifera* species, referred as the “wild” group, using boxplots, nested ANOVAs as well as Tukey’s test. Additionally, Student tests were undertaken for each of the four size parameters in order to compare cultivars and cultivated seedlings. The differentiation of shape among the four groups was appraised by a nested MANOVA carried out on the five first components of a PCA performed on the 64 Fourier coefficients. An LDA was undertaken on both size and shape variables on all samples except the four uncultivated date palms, in order to distinguish two groups: cultivated date palms and “wild” *Phoenix*. The distinction between them was assessed using the discriminant power computed with leave-one-out cross-validations as previously explained. The seeds from the four uncultivated date palms (two feral and two of unknown status) were included in the study as supplementary individuals. These individuals did not participate in the construction of the discriminant model but were projected onto the discriminant functions that were previously computed in order to predict which of the two groups they more probably belong to.

## Results

### Estimation of intra-individual seed sample size

**Fig 2:**
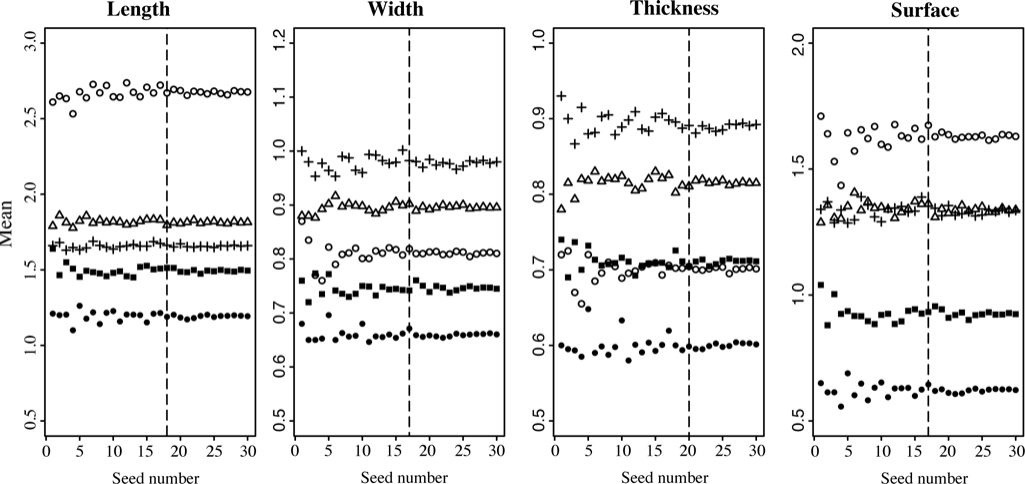
Mean of seed dimensions (in mm) calculated with an increasing number of randomly sampled seeds. White dot: 186_DEG2; Triangle: 1653_SYL20; Black square: 1876_RUP12; Black dot: 441_REC1; Cross: 1870_CAN62.

The seed number to analyse in order to stabilize the mean of the dimension parameters was quantified by randomly sampling 1 to 30 seeds in 5 individuals (Fig 2). The length mean appears stabilised, that is it stops fluctuating, with a minimum of 18 seeds. The width and surface means are stabilised with a minimum of 17 seeds. The thickness is stabilised with a minimum of 20 seeds. These results indicate that using 20 seeds is enough to describe the variability of size in *Phoenix* seeds, the same number as previously calculated to describe their shape [19]; the subsequent statistical analyses will thus be performed on 20 seeds per individual when available, that is a total of 1625 seeds (Table 1; Table 2).

### Description and comparison of seed size and shape in *Phoenix* species

Discrete measurements and Fourier coefficients for each seed can be found in S1 Appendix.

#### Difference in seed size between *Phoenix* species

Statistical properties on size of seeds (Fig 1) are given in Table 3 for each *Phoenix* species. Assumptions of data normality and homoscedasticity for the subsequent nested ANOVA were met (Shapiro-Wilk’s test: p-values < 0.05 and Bartlett's test: p-values < 0.05). The different *Phoenix* species display differences in size parameters (nested ANOVA, p-values < 2.10^−16^). The date palm displays the greatest seed length (Tukey’s test: p-value <0.05). It ranges from 1.12 to 3.16 cm with a mean of 2.08 cm while other *Phoenix* seeds have a maximum length of 2.02 cm, observed in *P*. *sylvestris*. It also displays the widest range of values for each size parameter, especially the length: the difference between the shortest and the longest seeds is greater than 2 cm while for other species this difference is lower than 1, except for *Phoenix reclinata* (Table 3). The variability in size, computed as the average dispersion of one hundred replicates of 20 randomly sampled seed points around the species centroids in a PCA space, is higher in *P*. *dactylifera* than in all other species (Table 4, Tukey’s test: p-value < 0.05) except *P*. *reclinata*.

**Table 3:** Mean, standard deviation, minimum (min) and maximum (max) of size parameters measured on each *Phoenix* species and groups derived from Tukey’s test.

	Number	Sample	Length (cm)				Width (cm)				Thickness (cm)				Surface (cm²)			
	of seeds	number	mean ± sd	min	max	Tukey's group(s)	mean ± sd	min	max	Tukey's group(s)	mean ± sd	min	max	Tukey's group(s)	mean ± sd	min	max	Tukey's group(s)
*P*. *acaulis*	62	4	1.01 ± 0.12	0.79	1.33	gh	0.58 ± 0.06	0.45	0.76	g	0.51 ± 0.07	0.35	0.68	g	0.47 ± 0.11	0.32	0.75	gh
*P*. *andamanensis*	13	1	1.41 ± 0.06	1.29	1.5	de	0.75 ± 0.03	0.69	0.81	d	0.66 ± 0.03	0.63	0.75	e	0.81 ± 0.06	0.68	0.91	e
*P*. *caespitosa*	60	3	1.18 ± 0.11	0.93	1.36	f	0.87 ± 0.07	0.71	1.01	c	0.77 ± 0.06	0.64	0.88	c	0.84 ± 0.17	0.52	1.18	de
*P*. *canariensis*	100	5	1.51 ± 0.16	1.25	1.78	c	0.95 ± 0.10	0.74	1.15	a	0.89 ± 0.09	0.69	1.09	a	1.17 ± 0.23	0.72	1.64	c
*P*. *dactylifera*	591	30	2.08 ± 0.43	1.12	3.16	a	0.85 ± 0.10	0.61	1.14	c	0.77 ± 0.10	0.49	1.06	c	1.34 ± 0.33	0.627	2.51	a
*P*. *loureiroi*	120	6	1.05 ± 0.13	0.8	1.32	g	0.61 ± 0.05	0.52	0.7	f	0.55 ± 0.04	0.46	0.64	f	0.51 ± 0.09	0.37	0.71	g
*P*. *paludosa*	70	4	0.99 ± 0.08	0.78	1.23	hi	0.69 ± 0.06	0.58	0.78	e	0.54 ± 0.05	0.45	0.64	f	0.52 ± 0.09	0.35	0.75	g
*P*. *pusilla*	100	5	0.95 ± 0.12	0.7	1.23	i	0.58 ± 0.08	0.44	0.71	g	0.52 ± 0.08	0.39	0.64	g	0.45 ± 0.11	0.25	0.68	h
*P*. *reclinata*	120	6	1.16 ± 0.31	0.73	1.9	f	0.70 ± 0.12	0.52	1.02	e	0.63 ± 0.12	0.44	0.94	e	0.68 ± 0.31	0.27	1.57	f
*P*. *roebelenii*	20	1	0.80 ± 0.03	0.75	0.86	j	0.43 ± 0.02	0.39	0.47	h	0.35 ± 0.02	0.32	0.37	h	0.27 ± 0.02	0.23	0.31	i
*P*. *rupicola*	69	4	1.44 ± 0.15	1.2	1.68	d	0.74 ± 0.05	0.67	0.88	d	0.71 ± 0.06	0.62	0.88	d	0.88 ± 0.15	0.66	1.16	d
*P*. *sylvestris*	200	10	1.65 ± 0.20	1.11	2.02	b	0.92 ± 0.10	0.73	1.13	b	0.83 ± 0.08	0.65	1.03	b	1.24 ± 0.22	0.67	1.71	b
*P*. *theophrasti*	100	5	1.33 ± 0.18	0.97	1.62	e	0.76 ± 0.08	0.59	0.93	d	0.72 ± 0.07	0.57	0.85	d	0.81 ± 0.18	0.5	1.17	e

**Table 4:** Variability of seed dimensions and shape within *Phoenix* species. It is calculated as the dispersion of seeds around the related species’ centroid in two PCA spaces obtained from size parameters (Size Var.) and 64 Fourier coefficients related to dorsal and lateral seed shapes (Shape Var.) using the rarefaction method. The values are the average over the mean distance between 20 randomly sampled seeds in one hundred replicates and the standard deviation over the one hundred replicates. The groups derived from Tukey’s test are given into parentheses.

Species	Number of seeds	Size Var. (Tukey’s group)	Shape Var. (Tukey’s group)
*Phoenix acaulis*	62	31.22 ± 6.59 (g)	284.91 ± 37.20 (c)
*Phoenix caespitosa*	60	39.15 ± 8.94 (fg)	238.57 ± 18.52 (d)
*Phoenix canariensis*	100	91.09 ± 20.14 (c)	224.32 ± 33.58 (e)
*Phoenix dactylifera*	591	128.02 ± 33.05 (b)	504.32 ± 76.44 (a)
*Phoenix loureiroi*	120	17.57 ± 3.25 (e)	205.71 ± 34.83 (e)
*Phoenix paludosa*	70	20.49 ± 5.21 (h)	178.87 ± 23.21 (f)
*Phoenix pusilla*	100	46.35 ± 6.12 (f)	151.48 ± 19.24 (g)
*Phoenix reclinata*	20	177.85 ± 43.14 (a)	269.30 ± 40.33 (c)
*Phoenix rupicola*	69	34.73 ± 6.90 (g)	171.17 ± 19.78 (fg)
*Phoenix sylvestris*	200	72.40 ± 22.30 (d)	432.62 ± 58.67 (b)
*Phoenix theophrasti*	100	57.43 ± 12.11 (e)	217.14 ± 34.99 (e)

#### Difference in seed shape between *Phoenix* species

**Fig 3:**
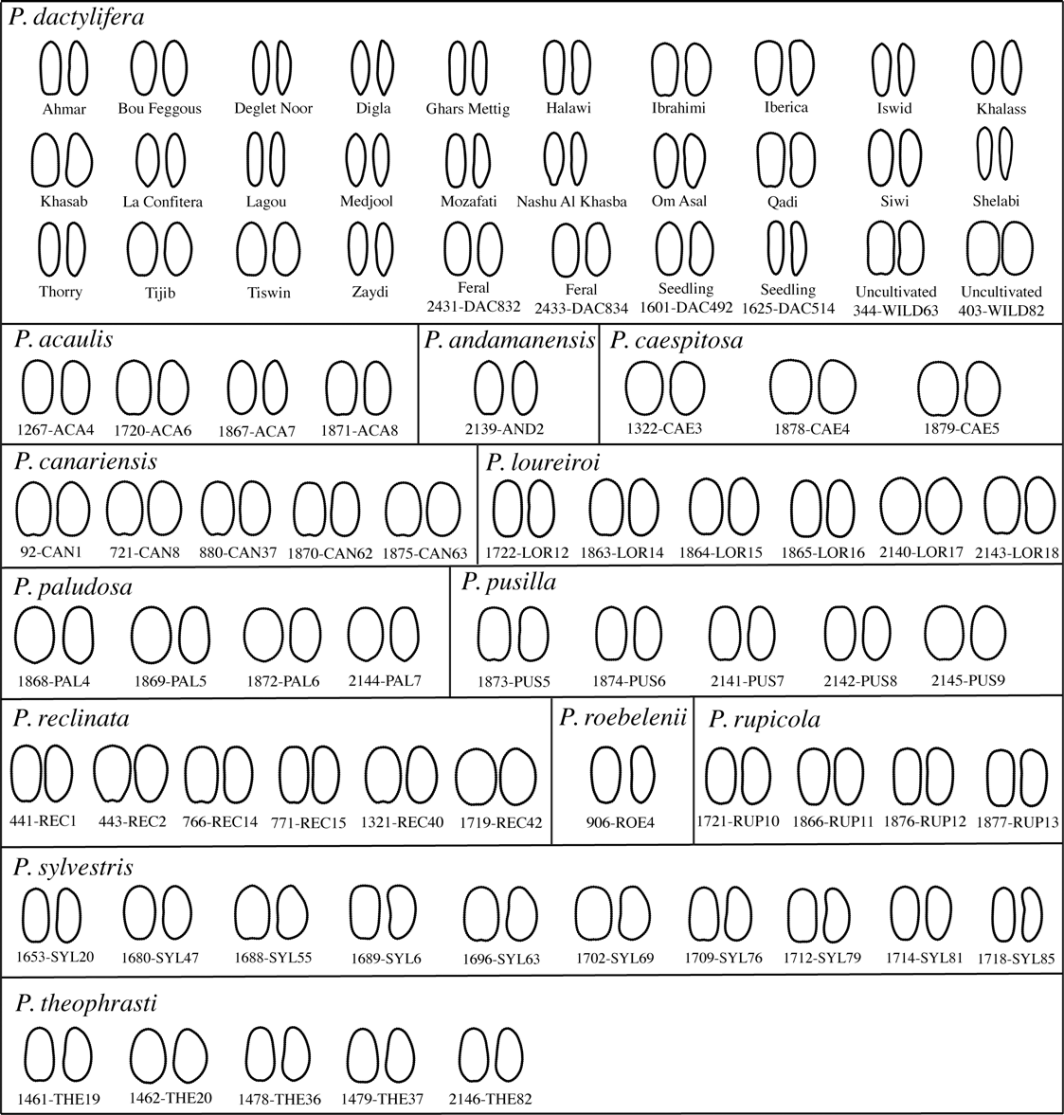
Reconstructed mean outlines of each individual included in this study using the inverse Fourier Transform method. Left: dorsal side; right: lateral side.

The mean outline of the 20 seeds for each individual was reconstructed using the inverse Fourier Transform method [56] (Fig 3). The existence of a seed shape difference among the different *Phoenix* species was tested with a nested MANOVA applied to the first five components over the components of a PCA analysis (explaining 51.38% of the variability) carried out on 64 Fourier coefficients after the homoscedasticity and the normality of the data were checked (Shapiro-Wilk’s test: p-values < 0.05 and Bartlett's test: p-values < 0.05). It indicates that a seed shape differentiation exists among the 13 *Phoenix* species included in this study (p-value < 0.01).

Like in the case of size parameters, we visually observe that the seed shape of date palms is greatly diversified compared to other *Phoenix* species’ (Fig 3) and this variability is reflected in the value of dispersion computed for each species (Table 4). This species appears more variable in shape than any other in the genus *Phoenix* (Tukey’s test: p-values<0.05). Within *Phoenix loureiroi*, *Phoenix rupicola*, *P*. *reclinata*, *P*. *sylvestris* and *P*. *dactylifera*, several morphotypes can be visually evidenced (Fig 3). Two sub-species of *P*. *loureiroi* are included here but the different morphotypes are not allocated to either of them so that the distinction between subspecies based on seed shape is not possible from these samples.

#### Characterizing *Phoenix* species based on seed size and shape

**Fig 4:**
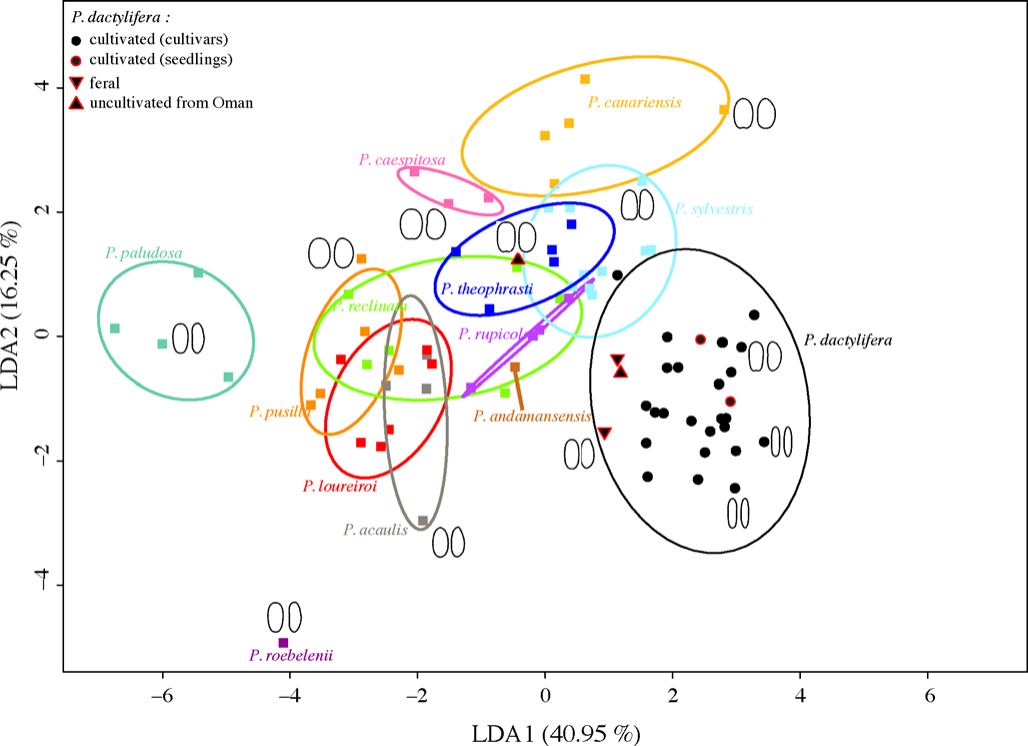
Linear Discriminant Analysis biplot of axis 1 (40.95% of variance explained) and axis 2 (16.25% of variance explained) performed on discrete measurements and 64 Fourier coefficients representing the dorsal and lateral seed shapes of 13 *Phoenix* species. Dorsal (left) and lateral (right) mean outlines are plotted for several individuals.

The LDA performed on both size and shape variables with the species being the discriminant parameter is plotted in Fig 4. The first axis represents the shape of seeds: on the left, species with rounded seeds like *Phoenix paludosa* are found, while at the right end is the date palm, displaying elongated seeds (Fig 4). The second axis (16.25%) is related to both size and shape. Indeed, *Phoenix* non *dactylifera* species are distributed upwards from the species with the smallest seeds (*P*. *roebelenii*) to the one with the largest (*Phoenix canariensis*), while the date palm, the species with the largest but elongated seeds, is found in the middle. Some species like *P*. *paludosa* and *P*. *canariensis* constitute a distinct group/cloud while the cloud of some species are overlapping like those of *Phoenix acaulis* and *P*. *loureiroi*. The addition of a third axis does not solve the overlapping problem. The cultivated date palm seeds constitute a distinguishable group although close to *P*. *sylvestris*. Seeds from uncultivated date palms are not found within the cultivated group but rather between the “wild” group and the cultivated group or in one case within the “wild” group. However, when the LDA is performed on shape only, the feral individuals are found within the points cloud of *P*. *dactylifera* while one of the uncultivated individuals form Oman is found within the “wild” *Phoenix* cloud (S1 Fig).

The mean percentage of correct allocation for each species computed with three different LDAs is given in Table 5. The size parameters alone do not allow a good distinction of species (36.4% on average). When using shape (dorsal and lateral sides combined), the average discriminant power increases substantially, reaching 68.8%. The addition of the dimension parameters to shape further increases the positive allocation for each species, with a mean of 79.5%. *P*. *paludosa*, *P*. *caespitosa*, *P*. *canariensis* and *P*. *roebelenii* appear as the easiest species to differentiate with a percentage of positive allocation above 90%. On the contrary, *P*. *acaulis*, *P*. *loureiroi* and *P*. *reclinata* are hardly differentiated from other *Phoenix* (percentage of positive allocation under 65%). *Phoenix dactylifera* is distinguished from other species at 87.8%. More specifically, when taking into account only cultivated date palms by discarding the uncultivated samples (feral from Egypt and uncultivated from Oman) this number reaches 92.9%.

**Table 5:** Discriminant power (in percentage) of the dimensions and/or shape of seeds in the genus *Phoenix*.

	Size	Outline	Size and outline
***P*. *acaulis***	17.7	36.2	48.1
***P*. *andamanensis***	6	64.4	82.9
***P*. *caespitosa***	29.9	89.9	94.5
***P*. *canariensis***	41.8	81.7	93
***P*. *dactylifera***	68.8	84.3	87.8
***P*. *loureiroi***	30.7	45.9	64.3
***P*. *paludosa***	74.7	97.2	97.4
***P*. *pusilla***	29.3	56.6	73.5
***P*. *reclinata***	16.1	44.3	50.2
***P*. *roebelenii***	59	84.6	95
***P*. *rupicola***	21.9	69.1	79.8
***P*. *sylvestris***	46.9	57.1	77.9
***P*. *theophrasti***	30.2	82.8	88.8
**Mean**	**36.4**	**68.8**	**79.5**

### Morphological features of cultivated date palm seeds compared to other *Phoenix* species

#### Correlation between size parameters

**Fig 5:**
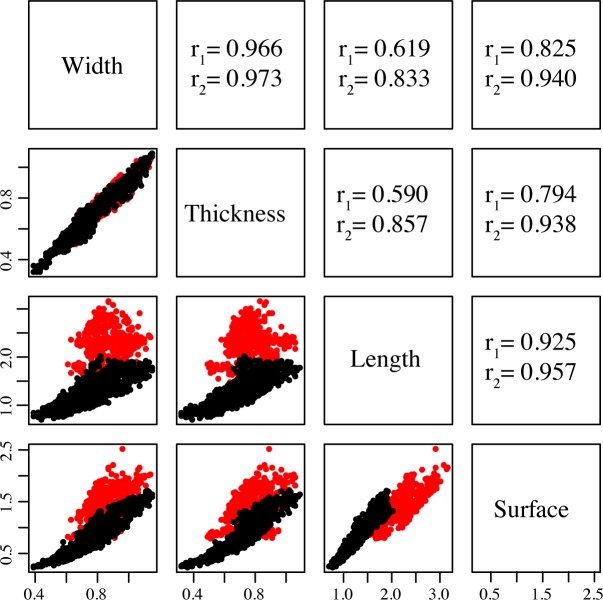
Correlation of size parameters. Red: *Phoenix dactylifera*, black: all *Phoenix* except *P*. *dactylifera*, in mm. Each pair of correlations is plotted on the lower left side while the coefficients of correlation *r* are given on the upper right-hand side: *r*_*1*_ corresponds to the correlation coefficients when all *Phoenix* species are included while *r*_*2*_ were calculated after discarding the date palm *P*. *dactylifera*.

Each pair of correlations between size parameters is significant (Fig 5; p-values << 0.01). When all the *Phoenix* species are included, the width and the thickness are highly correlated (r = 0.966) as well as the length and the surface of the dorsal side (r = 0.925). When discarding *P*. *dactylifera* from the correlation tests, both the thickness and the width were highly correlated with the length (r = 0.857 and r = 0.833 respectively).

#### Difference in seed size and shape in cultivars *versus* “wild” species

**Fig 6:**
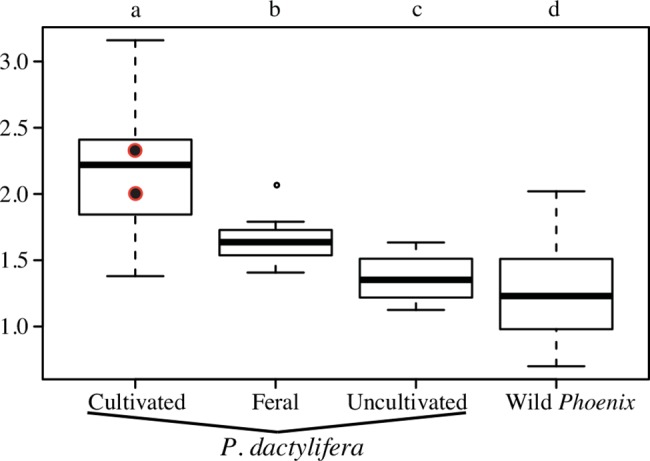
Comparison of seed length (in mm) between date palms from cultivated, feral and uncultivated populations of unknown status with wild *Phoenix*. The two black dots with red contour represent the average of seed length for the two cultivated date palm seedlings from India (1601_DAC492 and 1625_DAC514).

Seed length of cultivated date palms, feral date palms from Egypt, uncultivated date palms of unknown status from Oman and other *Phoenix* species (“wild”) is plotted in Fig 6. The boxplots related to the other three parameters measured may be found in S2 Fig. Within the cultivated date palm group, the seed size of seedlings (1601_DAC492 and 1625_DAC514) is comparable to the seed size of cultivars (Student tests: p-values >> 0.05). The nested ANOVAs performed on each parameter indicate that size is different according to the group they belong to (p-values < 0.01). Post-hoc Tukey’s tests between the 4 different statuses for the four size parameters are all significant (p-values < 0.05) except the width between uncultivated date palms from Oman and wild *Phoenix*. The cultivated date palm seeds display the greatest length, width, thickness and surface (Tukey’s test: p-value < 0.05). The feral date palm seeds from Egypt are smaller than the cultivated date palms seeds and larger than seeds of uncultivated individuals from Oman and of wild *Phoenix* species (Tukey’s test: p-value < 0.05). Uncultivated date palms from Oman have smaller seeds than cultivated and feral ones (Tukey’s test: p-value < 0.05). Additionally, the shape is also influenced by the status (nested MANOVA, p-value < 0.01). The LDA combining both size and shape features and performed to differentiate cultivated date palms from “wild” *Phoenix* provides a mean discriminant power of 94.7%: 90.5% of the cultivated seeds and 99.0% of the “wild” seeds are *a posteriori* positively allocated to their group. Within the two feral samples (individuals) from Egypt, 12 out of 40 seeds (30.0%) were allocated to the cultivated group while others were allocated to the “wild” group. The seeds from the supposed spontaneous date palm originating from Oman were all but one (97.5%) allocated to the “wild” group.

## Discussion

### Distinction of *Phoenix* species based on seed morphological features in the light of molecular data

While size seems weakly discriminant, the shape of seeds is highly distinctive for some species such as *P*. *paludosa*, almost a hundred percent discriminated from the other *Phoenix* species (Table 5). The combination of both seed size and shape provides a good rate of discrimination among *Phoenix* species (79.5%). However, some of them remain poorly discriminated. This is probably because of a high intraspecific variability (*P*. *reclinata*, Table 4) and/or a strong morphological similarity between species (*P*. *acaulis* and *P*. *loureiroi*). The high variability in size observed in the seeds of *P*. *reclinata* (Table 4) questions the existence of different ecotypes as previously proposed [21] and remains to be investigated with extensive sampling in relation to environmental parameters. Morphometrics of seeds thus appears as an efficient tool to differentiate most *Phoenix* species and should be considered for the identification of hybrids as previously stated [57].

Based on the analysis of chloroplastic sequences, *P*. *sylvestris* and *P*. *atlantica* are the closest relatives of the date palm *P*. *dactylifera* [18]. On the basis of seed size and shape results, *P*. *dactylifera* appears close to *P*. *sylvestris* (Figs 3 and 4), thus in agreement with genetic data. *Phoenix atlantica* is absent from the morphometric analysis thus its morphological proximity with the date palm remains to be assessed. This morphometric study thus corroborate genetic data [18,23,24] since these two methods allow to distinguish most *Phoenix* species and identify the date palm’s closest relatives.

### Seed peculiarities of date palms in the genus *Phoenix* and emphasis on the domestication syndrome

The variability in seed size and shape was assessed among equal-sized samples drawn from the different *Phoenix* species with a rarefaction method. The great variability of seed size and shape within the cultivated date palm was evidenced (Tables 3 and 4; Fig 3). The pattern of great phenotypic variability in cultivated species is well documented as a consequence of varietal diversification through space and time [58]. For the date palm, it may reflect its long-term history of cultivation associated with selection of traits (including fruit size and correlatively seed size), breeding and human-mediated diffusion [19].

The seeds from cultivated date palms are easily discriminated from those of wild other *Phoenix* species. On the one hand, seeds of “wild” species are smaller (Table 3; Fig 6) and rounded (Fig 3) and a strong correlation between their width/thickness and length was shown (Fig 5). On the other hand, seeds of cultivated date palms, whether they are seedlings or cultivars vegetatively propagated with offshoots, are longer (Table 3; Fig 6) and elongated (Fig 3), and they show no correlation pattern between thickness/width and length (Fig 5). These differences may be explained by divergent selection pressures leading to different patterns of morphological changes through time. Indeed, wild *Phoenix* are subject to a set of selection pressures including environmental constraints conditioning morphological evolution through time. Cultivated date palms are rather the subject of repeated strong human constraints related to cultivation practices that explain these particular phenotypes [19]. Moreover, seeds of wild *Phoenix* species and date palms growing without human influence (referred here as uncultivated date palms) seem to be submitted to constraints tending to minimize seed size and to standardize their phenotype. The canalization process, i.e. the ability of the organism to produce a constant phenotype despite genetic and/or environmental effects [59,60] leading in such palms to produce seeds with a similar phenotype (small and rather rounded), may be involved. As a corollary, the increase in seed length between wild progenitor and domesticated plant is a pattern observed for cereals [10,12], beans (e.g. soybean [61]) and fruit trees (e.g. Caimito [37], olive tree [35] and grapevine [36]). It has been shown to be correlated with an increase in fruit size [39–41]. Therefore, morphological changes or phenotypic trajectories from "wild" *Phoenix* species to cultivated date palm morphotype may be interpreted as a drastic shift related to selection pressures, and may be considered as a syndrome of domestication. As a result, we expect wild date palms to display small and rounded seeds; in addition, their length should be correlated to their width, thickness and surface.

The seeds of the four uncultivated individuals from Oman and Egypt appear smaller than that of the cultivated date palms and longer than that of “wild” *Phoenix* (Fig 6). Although the shift in seed size between wild and domesticated plants is related to artificial selection as stated before, the size of the seeds is also influenced by environmental and developmental factors as demonstrated for other models such as the olive tree [35]. Therefore, in the case of a search for distinctive criteria at the intra-specific level, seed size seems to be uninformative to distinguish feral from wild date palms as both may display small seeds as a result of constraining environmental conditions, while seeds from domesticated individuals develop large seeds as a consequence of selection and cultivation practices (irrigation and fertilization). In contrast, shape descriptors such as those used in this work were shown to be only slightly affected by environmental parameters and more powerful in a biosystematic point of view [19,34,38]. Feral date palms from Egypt display seeds presenting genuine affinity with cultivated date palms (Figs 3, 4 and 6). On the other hand, the uncultivated date palms from Oman show a morphology converging toward a wild *Phoenix* morphotype both in seed size and shape (Figs 3, 4 and 6). Thus, on the basis of morphometric data, we suggest that the individuals from Oman studied in this work may be true wild individuals even if some may have been introgressed by varieties cultivated in the region. Genetic analysis of these Oman populations are required to validate their wild status.

## Conclusion & Prospects

Through a morphometric approach combining traditional and geometric morphometrics, this study provides new and accurate insights into morphological changes of seed that occurred under domestication (i.e. syndrome of domestication). It allows us to discuss the possible existence of wild *Phoenix dactylifera* populations in the Middle East and thus the origins of the date palm. This study opens up exciting prospects for research and exploration of wild date palm populations that will represent a great challenge in preservation and conservation of biological resources.

In the future, predictive morphometric models applied to seed and previously validated by genetics will be applied to archaeological seeds such as those found in Miri Qalat and Shahi Tump, Pakistan [25]. A collaborative morphometric, genetic and archaeological approach will allow us to unravel the origins, the history, the historical biogeography and the evolution of the date palm through space and time.

This study includes the description of a pipeline of statistical analyses for (1) selecting the accurate number of seeds per sample, (2) quantifying and comparing seed size and shape and (3) studying the variability using a rarefaction method to equalize sample size. It could be applied to other crops and it thus constitutes a comprehensive methodology for the study of the domestication syndrome in seeds.

## Supporting Information

S1 Appendix Discrete measurements and elliptic Fourier coefficients of each seed included in the study. First sheet “Discrete measures” contains the measure of length, width, thickness (cm) and surface (cm^2^). Second sheet “Fourier coefficients” contains the Fourier coefficients obtained from the outline analyses based on Fourier method. Column C to AH contains the measures for dorsal view (name of the column suffixed with VD) and columns AI to BN contain the measures of the lateral side (suffixed with VL). In the name of those columns, A, B, C and D refers to the four coefficients of each harmonic and the following number to the number of the harmonic. For both sheets, first column (“Sample”) is the name of the sample; second column (“Seed”) is the number of the seed (D1 to D20 given that 20 seeds, when available, were analyzed for each sample) prefixed by the name of the sample.
(XLSX)

S2 Appendix Number of seeds to sample for the calculation of the intra-specific variability using the rarefaction method. (DOCX)

S1 Fig Linear discriminant analyses aiming to differentiate *Phoenix* species. Based on (A) four seed size parameters and (B) seed shape (64 normalized elliptic Fourier coefficients).
(PDF)

S2 Fig Comparison of seed width, thickness and surface between date palms from cultivated, feral and uncultivated populations of unknown status with wild *Phoenix* (in mm). (PDF)
