# Supplementary material for: The Domestication Syndrome in Phoenix dactylifera Seeds: Toward the Identification of Wild Date Palm Populations
Source: PLoS One. 2016 Mar 24;11(3):e0152394. doi: 10.1371/journal.pone.0152394 (PMC4807022; doi:10.1371/journal.pone.0152394)
Supplement: S2 Appendix — (DOCX) [file pone.0152394.s002.docx]

**S1 File. Number of seeds to sample for the calculation of the intra-specific variability using the rarefaction method.**

The implementation of the rarefaction method necessitates to choose a number of seeds to sample one hundred times so that the sample size will be the same in each different species for which the intra-specific variability is calculated.

This number should be at least twenty since this is the minimum number for the intra-individual variability (Terral et al., 2012; Fig. 2). We thus calculated the intra-specific variability using twenty to one hundred seeds using distances calculated in both a PCA performed on the 4 size parameters and the 64 Fourier coefficients related to dorsal and lateral seed shape.

Whether for size or shape, this test showed that the number of seeds sampled has no effect on the mean distance across the one hundred replicates (S1 File, Fig. 1-2).
