# Supplementary material for: The Domestication Syndrome in Phoenix dactylifera Seeds: Toward the Identification of Wild Date Palm Populations
Source: PLoS One. 2016 Mar 24;11(3):e0152394. doi: 10.1371/journal.pone.0152394 (PMC4807022; doi:10.1371/journal.pone.0152394)
Supplement: S1 Fig — Based on (A) four seed size parameters and (B) seed shape (64 normalized elliptic Fourier coefficients). (PDF) [file pone.0152394.s003.pdf]

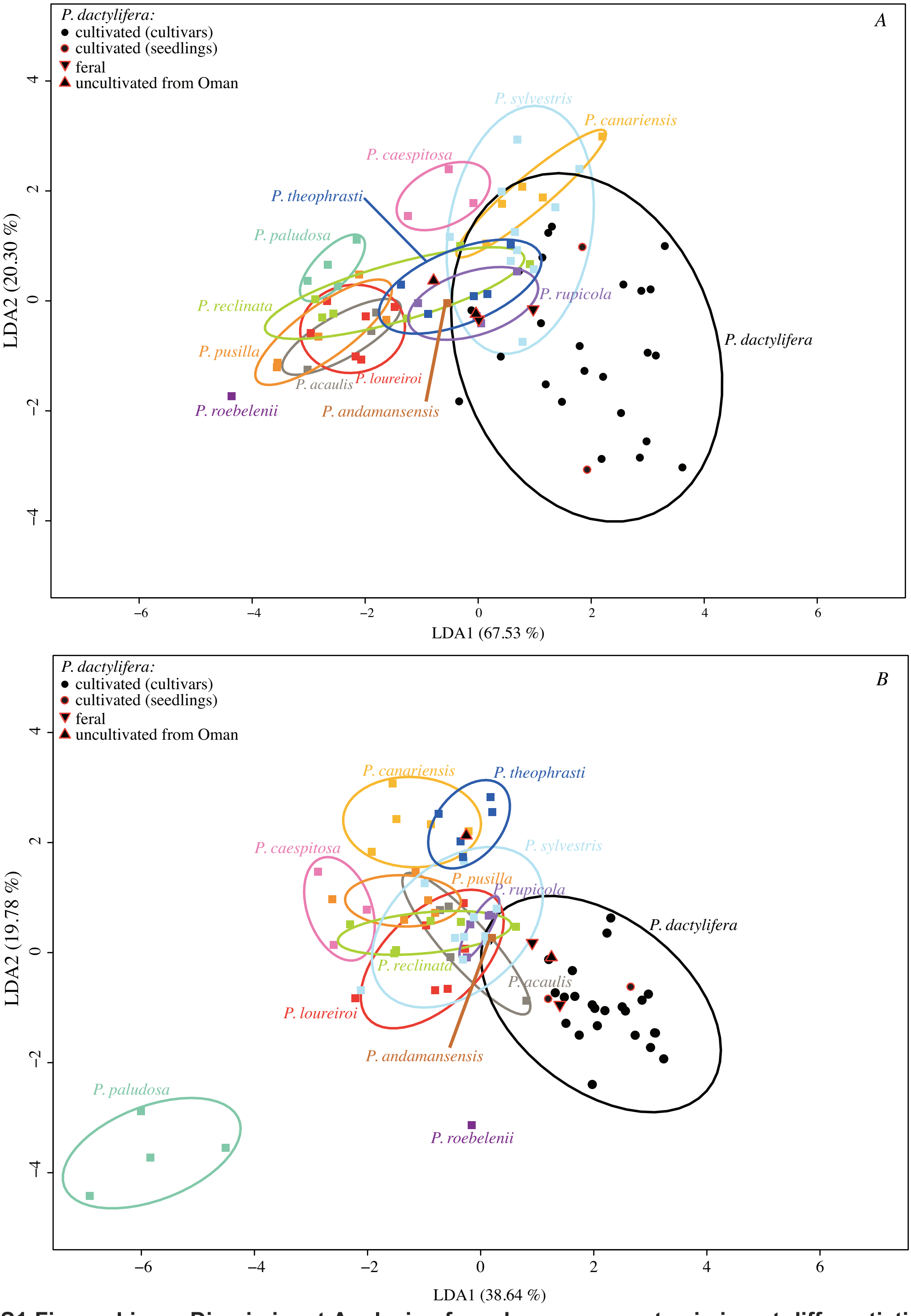

S1 Figure. Linear Discriminant Analysis of seed measurements aiming at differentiating *Phoenix* species. (A) four seed size parameters and (B) seed shape (normalized elliptic Fourier coefficients).
