# Supplementary material for: The Domestication Syndrome in Phoenix dactylifera Seeds: Toward the Identification of Wild Date Palm Populations
Source: PLoS One. 2016 Mar 24;11(3):e0152394. doi: 10.1371/journal.pone.0152394 (PMC4807022; doi:10.1371/journal.pone.0152394)
Supplement: S2 Fig — (PDF) [file pone.0152394.s004.pdf]

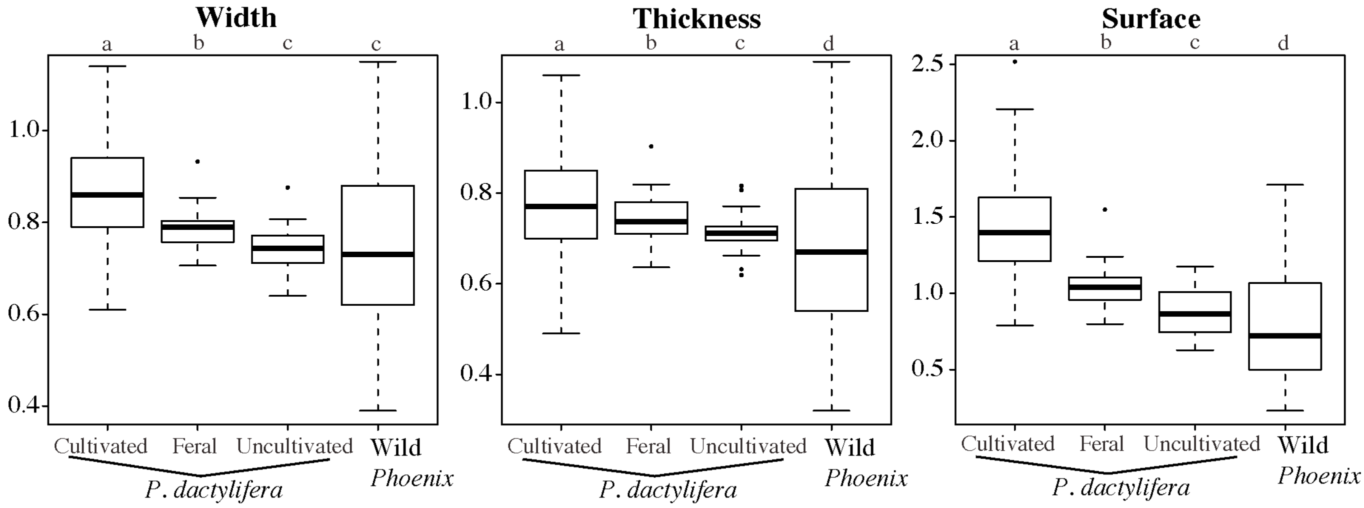

S2 Figure. Comparison of seed width, thickness and surface between date palms from cultivated, feral and uncultivated populations of unknown status with wild *Phoenix* (mm). The group derived from Tukey's test is displayed on top of each group's boxplot.
